# Rapid Detection of Quinolone Resistance Mutations in *gyrA* of *Helicobacter pylori* by Real-Time PCR

**DOI:** 10.3390/pathogens11010059

**Published:** 2022-01-03

**Authors:** Franziska Haumaier, Anna Schneider-Fuchs, Steffen Backert, Michael Vieth, William Sterlacci, Birgitta M. Wöhrl

**Affiliations:** 1 Lehrstuhl Biochemie IV—Biophysikalische Chemie, Universität Bayreuth, Universitätsstraße 30, 95447 Bayreuth, Germany; franzihaumaier@googlemail.com; 2 Institut für Pathologie, Klinikum Bayreuth, Preuschwitzer Straße 101, 95445 Bayreuth, Germany; anna.schneider-fuchs@klinikum-bayreuth.de (A.S.-F.); Vieth.LKPathol@uni-bayreuth.de (M.V.); 3 Department Biologie, Lehrstuhl Für Mikrobiologie, Friedrich-Alexander Universität Erlangen-Nürnberg, Staudtstraße 5, 91058 Erlangen, Germany; steffen.backert@fau.de; 4 Institut für Pathologie, Friedrich-Alexander Universität Erlangen-Nürnberg, Klinikum Bayreuth, Preuschwitzer Straße 101, 95445 Bayreuth, Germany; 5 Bayreuther Zentrum für Molekulare Biowissenschaften (BZMB), Universität Bayreuth, Universitätsstraße 30, 95447 Bayreuth, Germany

**Keywords:** *Helicobacter pylori*, antibiotic resistance, quinolone resistance, QRDR mutation diagnosis, FRET, *gyrA*, RT-PCR

## Abstract

The treatment of infections by the gastric pathogen *Helicobacter pylori* (*H. pylori*) has become more difficult due to increased rates of resistances against various antibiotics. Typically, atriple therapy, employing a combination of at least two antibiotics and a proton pump inhibitor, is used to cure *H. pylori* infections. In case of first-line therapy failure, quinolones are commonly applied in a second-line therapy. To prevent second-line treatment failures, we developed an improved method to detect the most common quinolone-resistance mutations located in the quinolone-resistance-determining region (QRDR) of the bacterial *gyrA* gene. Biopsy material from the gastric mucosa of infected patients was used to identify quinolone-resistant strains before the onset of drug administration. Two different wild-type and six mutant QRDR sequences were included. Melting curve analyses were performed with corresponding *gyrA* plasmid DNAs using a real-time polymerase chain reaction (RT-PCR) assay. By applying a combination of only two different fluorescent probes, this assay allows wild-type sequences to be unambiguously distinguished from all known mutant QRDR sequences of *H. pylori*. Next, the T_m_ values of patient DNAs were established, and the genotypes were confirmed by sequencing. Thus, quinolone-resistant *H. pylori* strains can be easily and quickly diagnosed before treatment, which will help to avoid the administration of ineffective drug regimes.

## 1. Introduction

The Gram-negative bacterium *Helicobacter pylori* (*H. pylori*) infects the gastric mucosa and is associated with different human diseases, e.g., chronic active gastritis, peptic ulcer disease, gastric cancer or mucosa-associated lymphoid tissue (MALT)-lymphoma [1]. In Germany, *H. pylori* infections are commonly treated first-line by a triple therapy consisting of two different antibiotics, usually amoxicillin and clarithromycin or metronidazole, and a proton pump inhibitor (PPI) [2]. However, this standard first-line therapy for the eradication of *H. pylori* faces a serious problem due to the increasing rates of resistance against these antibiotics, which may result in therapy failure. Thus, a quadruple therapy consisting of bismuth in combination with amoxicillin, a PPI and a quinolone, e.g., levofloxacin, is now often recommended [3,4].

Quinolones target the DNA gyrase, which is an essential enzyme in bacterial replication that can create negative and positive supercoils in DNA by transiently introducing double-strand breaks in an ATP-dependent reaction [5]. DNA gyrase is heterotetrameric consisting of two A and two B subunits, respectively. The A subunit contains a tyrosine residue in the active center that covalently binds the newly generated 5′ termini of DNA during the cleavage reaction, while the B subunit harbors the ATPase domain responsible for DNA cleavage and ligation. Quinolones were first reported to target the A subunit of the DNA gyrase in *H. pylori* [6]. However, it has been also suggested that mutations in the B subunit may contribute to quinolone resistance [7,8,9]. In particular, resistance against quinolones can be achieved by single point mutations in the so-called quinolone resistance-determining region (QRDR) of the *gyrA* gene of *H. pylori* [10,11,12]. The mutations conferring resistance result in specific amino acid exchanges at codons 87 and/or 91 in QRDR, which, in turn, lead to a weaker binding of the antibiotic. The most common amino acid exchanges appear to be N87K and D91G, D91N and D91Y (Table 1) [13,14,15]. The N87K mutation alone confers high resistance to levofloxacin and gatifloxacin [7]. Depending on the geographical region, in which the isolates were obtained, additional but rarely occurring amino acid exchanges have been detected, i.e., N87I, N87R, D91M, D91C, D91A, and N87H in combination with D91M [16,17]. However, the contribution of these mutations to antibiotic resistance has not been tested in all cases.

A common method to examine resistance to quinolone is via hybridization probes on stripes. This procedure is used in the commercial kit GenoType HelicoDR from Hain Lifesciences (Nehren, Germany). Despite the advantageous suitability of this kit for cell culture testing, it requires, in addition to a thermal cycler, an incubator and an apparatus for the visual evaluation of the colour reactions on the stripes. Moreover, this test takes more than five hours to complete. Another resistance-determining technique is the whole-genome sequencing of *H. pylori* via the Next Generation Sequencing (NGS) methodology. NGS is especially useful in the identification of new resistance mutations. However, this approach is very expensive and time-consuming for routine examinations [18]. Above all, for the treatment of *H. pylori* infections, it is important to be able to distinguish between wild-type (WT) and mutant (MT) QRDR sequences. These requirements can be met by an RT-PCR-based assay, which can be performed in only a few hours. Furthermore, compared to any dot blot hybridization method, the sensitivity obtained using real-time polymerase chain reaction (RT-PCR) is much higher, because DNA amplification can detect very low copy numbers of the specific DNA sequence.

In this work, we developed an assay based on melting curve analyses using RT-PCR to distinguish between the known WT and the most prevalent MT QRDR sequences (Table 1) [13,14,15]. In contrast to previous approaches, by cultivating the bacteria to determine the resistance pattern (E-test), which usually takes about five days, genotyping with RT-PCR can provide consistent results within one day, thus saving time, money and additional biopsy procedures for culture. This method allows for the reliable identification of *H. pylori* strains in patients, which are resistant to quinolones using an RT-PCR assay based on Förster resonance energy transfer (FRET), and the determination of different melting point temperatures (T_m_) of fluorescently labeled probes that are complementary to the relevant QRDR sequence. The assay only requires a set of three different probes, one WT sequence and two mutant sequences to reliably determine if resistant *H. pylori* is present or not. Our method provides a rapid and cost-effective new tool for the improved detection of all prevalent resistance-associated mutations against quinolones in the QRDR and, in addition, can discriminate between MT and WT sequences. Thus, the optimal combination of antibiotics for the patient can be determined before treatment.

## 2. Results and Discussion

The aim of this study was to develop a fast and low-cost assay, which can unambiguously distinguish between *H. pylori* strains from patient biopsies that are sensitive or resistant to quinolone antibiotics. Using this strategy, inefficient treatment of patients can be avoided with savings in time, money and additional biopsy procedures for culture. A previously published assay detected *gyrA* mutations in *H. pylori* by allele-specific PCR [19]. However, this method requires the application of a set of eight different allele-specific primers. After PCR amplification, the PCR products have to be visually analyzed by agarose gel electrophoresis and are distinguished according to their size.

**Figure 1:**
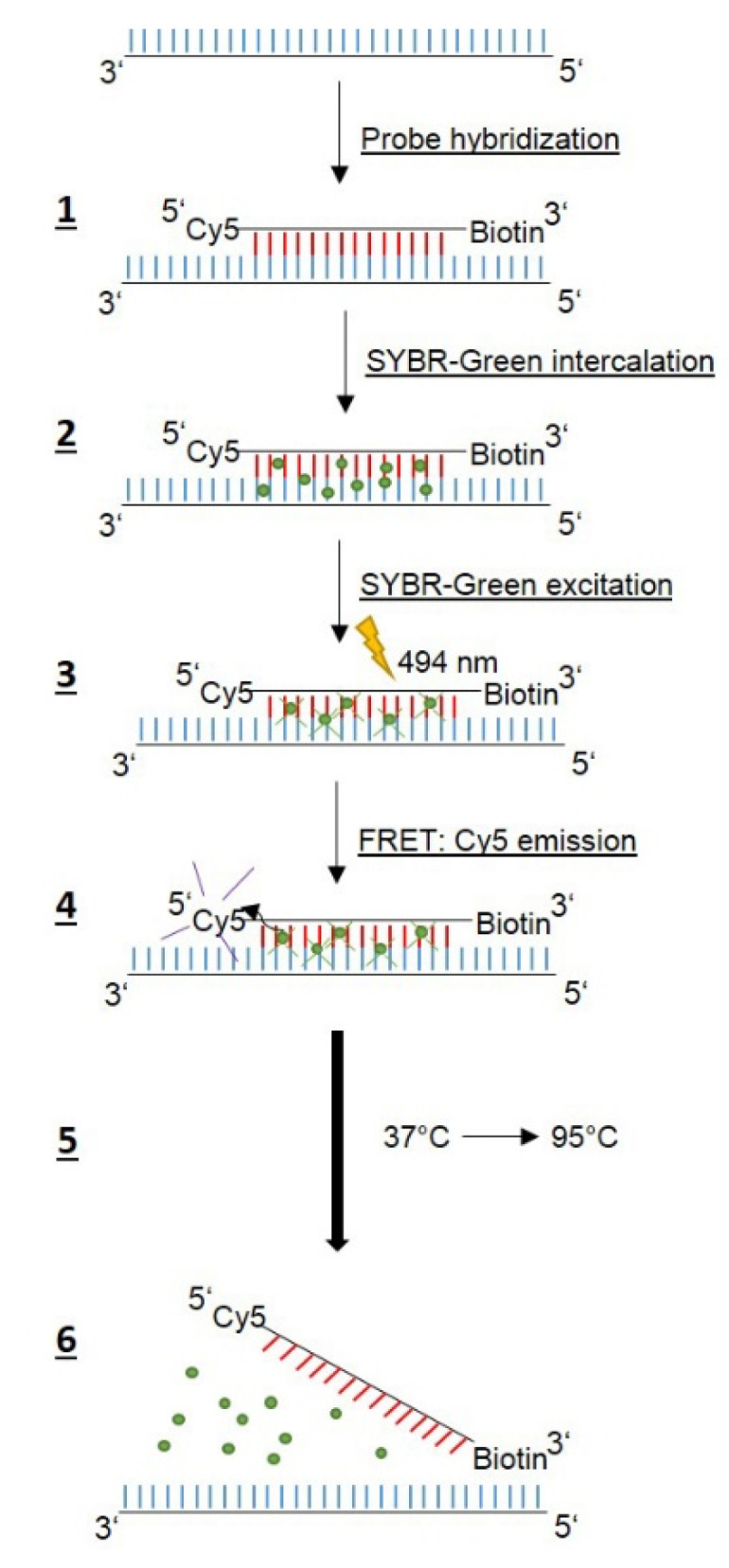
Schematic presentation of the sequence of events performing a melting curve analysis by RT-PCR with a Cy5 labeled hybridization probe and SYBR-green. Six individual steps were defined as indicated. For more details, see text and Table 2, Table 3 and Table 4.

A RT-PCR assay, developed by Glocker and Kist [14], aimed to detect WT and MT QRDR sequences bya Förster resonance energy transfer (FRET)-based RT-PCR assay, using a labeled anchor probe in combination with a labeled mutation probe for each codon. The assay established here combines that approach with a method used for the detection of clarithromycin resistance in *H. pylori,* as reported by Schabereiter-Gurtner et al. [20]. Only two fluorescent probes, specific to the mutant genotypes corresponding to MT1 and MT3, respectively (Table 1), are needed, but no anchor probes, since here FRET is based on the intercalation and excitation of SYBR-Green in the amplified DNA, followed by emission via the Cyanine5 (Cy5) label of the probe (Figure 1).

To find reliable assay conditions, we first generated plasmid DNAs harboring two defined WT sequences (WT1 and WT2) and six MT QRDR sequences (MT1 to MT6) as shown in Table 1 and Table 2, respectively. We decided not to use different fluorescent labels, which would allow two probes to be used at the same time to avoid misinterpretations, since all probes bind to the same region. A single-stranded DNA probe complementary to the QRDR WT1 sequence, labeled with Cy5 at the 5′ end and biotin at the 3′ end to prevent polymerization, was used for melting curve analyses (Table 3). SYBR-green, a dye intercalating into double-stranded DNA was added to the reaction mix. RT-PCR and subsequent melting curve analysis (Table 4) was performed according to the scheme shown in Figure 1. The Cy5 WT1 probe binds specifically to the region harboring codons 87 and 91 of the amplified DNA, while SYBR-green intercalates into the double-stranded DNA. The excitation of SYBR-green at 494 nm results in FRET to the Cy5 label, and Cy5 emission can be detected at 670 nm. Applying a temperature-gradient (37 °C to 95 °C) leads to dissociation of the Cy5 probe at a specific temperature and, thus, the FRET signal abates. Accordingly, mutations in the QRDR template DNA will give rise to lower T_m_ values due to the weaker binding of the Cy5 probe, which is 100% complementary to only the WT1 QRDR.

**Figure 2:**
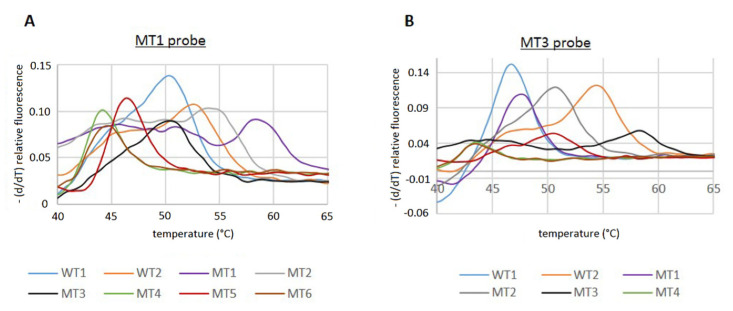
Melting curve analyses of WT and MT QRDR sequences by RT-PCR. Analyses were performed according to the protocol shown in Table 4 with the MT1 probe (**A**) or MT3 probe (**B**).

Using the Cy5 WT1 probe (Table 3), corresponding to the WT1 QRDR sequence, resulted in a high background noise, which made analysis of the melting curves difficult, and no signal could be detected for MT3 (data not shown). Thus, we designed new Cy5 probes corresponding to MT1 and MT3 (Table 3) and performed RT-PCR reactions to determine the T_m_-values. With both probes, the two WT sequences, as well as all MT QRDR sequences (Table 1), could be detected and exhibited different T_m_ values (Figure 2).

However, since the T_m_ values of some of the MT PCR products were very similar compared to the WT, it was not possible to unambiguously distinguish between all MT and WT sequences with just one of the Cy5 probes. Using the MT3 probe, no discrimination of the T_m_ of the WT1/MT1, MT2/MT5, and MT4/MT6 was possible. However, the MT1 probe allowed us to distinguish between the WT1 and MT1 sequence, but the discrimination of WT2/MT2, WT1/MT3, and MT4/MT6 could not be achieved (Figure 2; Table 5).

Nevertheless, our setup allowed for a general discrimination between WT and MT sequences. Since only patients infected with *H. pylori* WT strains, which are susceptible to quinolones, can be successfully treated with the antibiotic, it is sufficient to be able to separate the WT sequences from all MT sequences. Thus, by using our two MT Cy5 probes in parallel, it is possible to assign a sequence either to a WT or MT phenotype, respectively. If a patient sample is tested with the MT1 probe and a T_m_ of ca. 50 °C is determined, it will not be clear whether the *H. pylori* strain harbors the WT1 or MT3 QRDR sequence (Table 5). Thus, a second RT-PCR reaction with the MT3 probe can be performed for identification. However, with the MT3 probe, the WT1 will have a T_m_ of 46.86 °C, the T_m_ of the MT3 genotype will be increased to 58.2 °C due to the 100% complementarity of the MT3 probe. The same procedure can be applied if T_m_ = 52.7 °C, and no distinction between WT2 and MT2 can be made, since the T_m_ values of the two sequences differ by 4 °C when the MT3 probe is used. On the other hand, no distinction between WT1 and MT1 can be made if only the MT3 probe is used. However, by applying the MT1 probe, the T_m_ values are easily distinguishable (49.99 °C vs. 58.49 °C).

**Figure 3:**
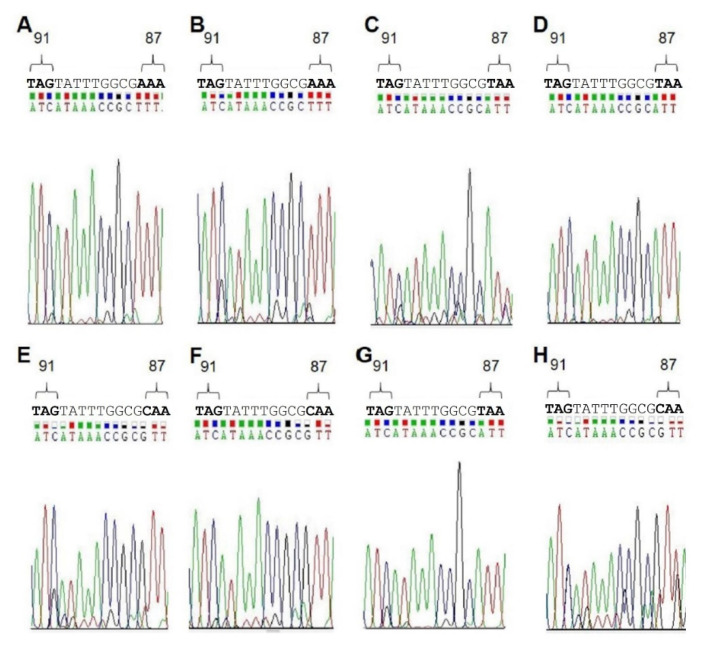
Sequencing data obtained from DNA in gastric biopsies of *H. pylori*-positive patients. Eight DNA samples (**A**–**H**) used for melting curve analysis were sequenced with the *gyrA*-RP as sequencing primer (Table 3). The relevant region of the QRDR harboring codons 87 and 91 is shown.

Finally, to further examine the validity of our results, we analyzed DNA isolated from biopsies taken from patients with known *H. pylori* infections. Each sample was tested with the MT1, as well as with the MT3 probe. In addition, to confirm the results obtained by melting curve analyses, the patient DNAs were sequenced to determine the QRDR genotype (Table 6, Figure 3). All sequencing data were in agreement with the genotype determined by RT-PCR (Table 6, Figure 3). As negative controls, and to determine the specificity of our test, we isolated biopsy DNA from six patients known not to be infected with *H. pylori* and performed RT-PCR with the QRDR primers. As expected, no QRDR sequence could be detected in all cases (Table 6, bottom, Supplementary Data, Figure S1A,B), which confirms the good specificity of this test.

## 3. Material and Methods

*Plasmids.* The *gyrA* QRDR sequence of two antibiotics-susceptible clinical WT strains were utilized, WT1 (GenBank accession no. CP026515.1) and WT2 (GenBank accession no. CP026515.1). The two WT sequences differ only in the codon at position 87 (Table 1, top). The WT1 *gyrA* sequence was amplified by PCR with the FP and RP GyrA WT1 primers (Table 2) using sequenced patient DNA as a template, and cloned into the vector pUC57 via the restriction sites XbaI and BamHI (Genscript, Piscataway, NJ, USA). WT2 and six QRDR mutations (named MT1 to MT6) were introduced by site-directed mutagenesis in the WT1 sequence (Table 1). The primers for mutagenesis shown in Table 2 were applied according to the Quick Change mutagenesis protocol (Agilent Technologies, Santa Clara, CA, USA) and confirmed by standard sequencing (GATC Biotech AG, Konstanz, Germany). Plasmids were propagated in *Escherichia coli* strains XL1-Blue or Top10 and isolated using the QIAprep Spin Miniprep Kit (Qiagen, Hilden, Germany).

Patient DNA isolation, amplification and sequencing. DNA of patients infected with *H. pylori* was isolated from formalin fixed, paraffin embedded (FFPE) gastric biopsies (ethics statement number: 322_21 Bc, University of Erlangen, Germany) using the Maxwell^®^ 16 LEV Blood Kit (Promega GmbH, Mannheim, Germany). Usually, six thin sections of 2 µm thickness were used for DNA extractions. The concentration was measured by UV-Vis spectroscopy at 260 nm. Approximately 200–400 ng of isolated DNA was used to amplify the QRDR sequence by PCR using the primers gyrA-FP and gyrA-RP (Table 3) with the PCR program depicted in Table 4 without the melting curve step. To avoid misinterpretation of the results, lower DNA concentrations are not recommended. The amplified QRDR fragments were sequenced using gyrA-RP as a sequencing primer (GATC Biotech AG, Konstanz, Germany).

Melting curve analyses. All plasmid and patient DNAs were analyzed by melting curve analyses. Primers and probes (metabion GmbH, Planegg/Steinkirchen, Germany) used are shown in Table 3. The reaction mix contained 0.625 µM forward primer (FP), 0.19 µM reverse primer (RP), 0.5 µM probe, 3 mM MgCl_2_, 2 µL LightCycler FastStart DNA Master SYBR green I (Roche Diagnostics, Mannheim, Germany) and 40 ng of template DNA in 20 µL reactions. Samples were analyzed using a Cobas Z 480 Analyzer (Roche Diagnostics, Mannheim, Germany) according to the RT PCR program shown in Table 4. The results were evaluated with the *LightCycler^®^ 480 software 1.5.1* (Roche Diagnostics, Mannheim, Germany). Several independent melting curve analyses were performed for each QRDR sequence and the average of the melting temperature (T_m_), as well as the standard deviation (S) were estimated according to Equations (1) and (2):

(1) $$\bar{T_{m}}=\frac{\left(\sum^{}T_{m}\right)}{n}$$

(2) $$S=\sqrt{\frac{\sum_{n}{{(T}_{m}-\bar{T_{m}})}^{2}}{n}}$$

n, number of measurements; T_m_, melting temperature; $\bar{T_{m}}$, average of melting temperature of n measurements; S, standard deviation.

## 4. Conclusions

Our results demonstrate that the FRET-based melting curve analysis using SYBR-Green in combination with two Cy5 labeled probes provides a valuable and reasonably priced tool, since the assay can be performed by various commercially available RT-PCR instruments, which are able to detect the Cy5 signal. If necessary, the method can be extended to include rare mutations by determining the corresponding melting temperature. It can reliably distinguish between WT and MT QRDR sequences, which is necessary to efficiently treat patients with quinolones.

## Figures and Tables

**Table 1:** Sequence of wild-type and mutant codons of gyrA QRDR.

Genotype	*gyrA*-Codon 87 (aa) *	*gyrA*-Codon 91 (aa)
**WT1**	AAC (Asn)	GAT (Asp)
**WT2**	AAT (Asn)	GAT (Asp)
**MT1**	AAA (Lys)	GAT (Asp)
**MT2**	AAG (Lys)	GAT (Asp)
**MT3**	AAT (Asn)	GGT (Gly)
**MT4**	AAC (Asn)	TAT (Tyr)
**MT5**	AAT (Asn)	AAT (Asn)
**MT6**	AAC (Asn)	AAT (Asn)

* The corresponding amino acid (aa) is shown in parenthesis.

**Table 2:** Cloning and mutagenesis primers.

Primer	Sequence *
**FP GyrA WT1**	5′-ATG CAT GAA TTA GGT CTT ACT-3′
**RP GyrA WT1**	5′-TTC TTC ACT CGC CTT AGT CAT-3′
**FP GyrA WT2**	5′-C CAC CCC CAT GGC GAT **AAT** GCG GTT TAT GAT GCA CTA G-3′
**RP GyrA WT2**	5′-C TAG TGC AT**C** ATA AAC CGC **ATT** ATC GCC ATG GGG GTG G-3′
**FP GyrA MT1**	5′-C CAC CCC CAT GGC GAT **AAA** GCG GTT TAT GAT GCA CTA G-3′
**RP GyrA MT1**	5′-C TAG TGC AT**C** ATA AAC CGC **TTT** ATC GCC ATG GGG GTG G-3′
**FP GyrA MT2**	5′-C CAC CCC CAT GGC GAT **AAG** GCG GTT TAT GAT GCA CTA G-3′
**RP GyrA MT2**	5′-C TAG TGC AT**C** ATA AAC CGC **CTT** ATC GCC ATG GGG GTG G-3′
**FP GyrA MT3**	5′-C CAC CCC CAT GGC GAT AAT GCG GTT TAT **GGT** GCA CTA G-3′
**RP GyrA MT3**	5′-C TAG TGC **ACC** ATA AAC CGC ATT ATC GCC ATG GGG GTG G-3′
**FP GyrA MT4**	5′-GGC GAT AAC GCG GTT TAT **TAT** GCA CTA GTG AGA ATG G-3′
**RP GyrA MT4**	5′-C CAT TCT CAC GAT TGC **ATA** ATA AAC CGC GTT ATC GCC-3′
**FP GyrA MT5**	5′-C CAC CCC CAT GGC GAT AAT GCG GTT TAT **AAT** GCA CTA G-3′
**RP GyrA MT5**	5′-C TAG TGC **ATT** ATA AAC CGC ATT ATC GCC ATG GGG GTG G-3′
**FP GyrA MT6**	5′-GGC GAT AAC GCG GTT TAT **AAT** GCA CTA GTG AGA ATG G-3′
**RP GyrA MT6**	5′-C CAT TCT CAC GAT TGC **ATT** ATA AAC CGC GTT ATC GCC-3′

* The mutated bases are shown in bold. FP, forward primer; RP, reverse primer.

**Table 3:** Sequences of hybridization probes and primers used for RT-PCR.

Oligo	Sequence
**FP GyrA WT1**	5′-ATG CAT GAA TTA GGT CTT ACT-3′
**RP GyrA WT1**	5′-TTC TTC ACT CGC CTT AGT CAT-3′
MT3 probe *	5′-Cy5-**ACC** ATA AAC GGC ATT ATC GCC A-Biotin-3′
**MT1 probe**	5′-Cy5-AT**C** ATA AAC GGC **TTT** ATC GCC A-Biotin-3′
**WT1 probe**	5′-Cy5-TGC ATC ATA AAC CGC GTT ATC G-Biotin-3′

* Sequence taken from [14]. The mutated bases are shown in bold.

**Table 4:** RT-PCR program for melting curve analysis.

	Temperature (°C)	Time (s)	Number of Cycles	Temperature Increase (°C/s)
**Initial DNA denaturation**	95	600	1	4.4
**DNA denaturation**	95	5	70	4.4
**Hybridization**	65	10	70	2.2
**Primer elongation**	72	6	70	4.4
**Melting of double strands**	95	1	1	4.4
**Binding of probe**	37	1	1	2.2
**Melting curve**	37–95	967	1	0.6
**Cooling**	4	1	∞	4.4

**Table 5:** T_m_-values of MT1 and MT3 probes.

Genotype	T_m_ MT1 Probe (°C)	T_m_ MT3 Probe (°C)
**WT1**	49.99 ± 0.184	46.86 ± 0.139
**WT2**	52.58 ± 0.057	54.79 ± 0.625
**MT1**	58.49 ± 0.099	47.61 ± 0.179
**MT2**	53.43 ± 0.629	50.72 ± 0
**MT3**	50.16 ± 0.201	58.20 ± 0.165
**MT4**	44.26 ± 0.114	43.61 ± 0.132
**MT5**	46.42 ± 0.120	50.54 ± 0.231
**MT6**	44.62 ± 0.057	43.59 ± 0.206

The mean values including the standard deviation of three independent experiments are shown. The different shades of gray (dark, medium, light) indicate T_m_ pairs that cannot be distinguished using only the MT1 or MT3 probe, respectively.

**Table 6:** Assignment of patient DNA samples by melting curve analyses and DNA sequencing.

DNA Sample	T_m_ MT1 Probe (°C)	T_m_ MT3 Probe (°C)	Assignment	DNA Sequencing
**A**	58.9	47.8	MT1	MT1
**B**	59.4	48.8	MT1	MT1
**C**	53.3	56.2	WT2	WT2
**D**	53.9	54.7	WT2	WT2
**E**	48.7	46.4	WT1	WT1
**F**	51.5	48.2	WT1	WT1
**G**	53.5	56.2	WT2	WT2
**H**	51.5	47.9	WT1	WT1
controls *	/	/	/	/

* six patient samples without known *H. pylori* infection were used as controls.

## Data Availability

Data were taken from the archive of the Institute of Pathology and stored there according to national law. Data for this study were anonymised in accordance with data protection laws and local regulations.

## Supplementary Materials

The following are available online at https://www.mdpi.com/article/10.3390/pathogens11010059/s1, Figure S1: Melting curve analyses with the MT1 and MT 3 probes and DNA from patients without *H. pylori* infection.

## Abbreviations

**Abbreviation**	**Full Name**
Cy5	Cyanine5
FRET	Förster resonance energy transfer
FP	forward primer
*H. pylori*	*Helicobacter pylori*
MT	mutant
QRDR	Quinolone-resistance-determining region
RP	reverse primer
RT-PCR	real-time polymerase chain reaction
T_m_	melting point
WT	wild-type
