# Supplementary material for: Rapid Detection of Quinolone Resistance Mutations in gyrA of Helicobacter pylori by Real-Time PCR
Source: Pathogens. 2022 Jan 3;11(1):59. doi: 10.3390/pathogens11010059 (PMC8781667; doi:10.3390/pathogens11010059)

Supplementary Data

Figure S1: Melting curve analyses with the MT1 and MT 3 probes and DNA from patients without *H. pylori* infection. RT-PCR specificity analyses were performed with the probes MT1 **(A)** and MT3 **(B)** and DNA from six patients without *H. pylori* (NEG1-NEG6), an additional negative (NEG) control lacking DNA, and a non-mutated QRDR sequence of a *H. pylori* as a positive control (POS).

A

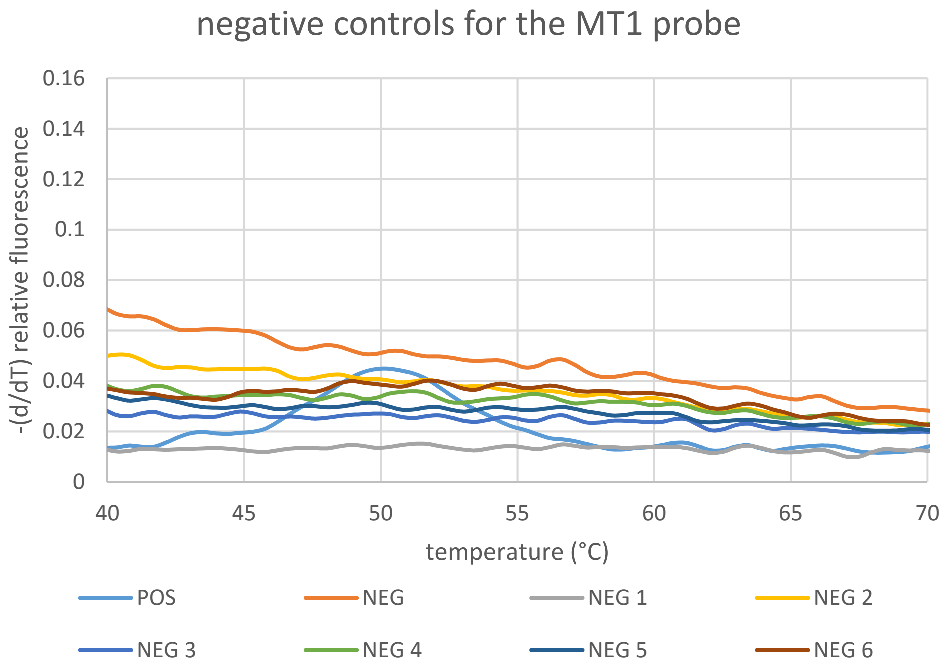

B

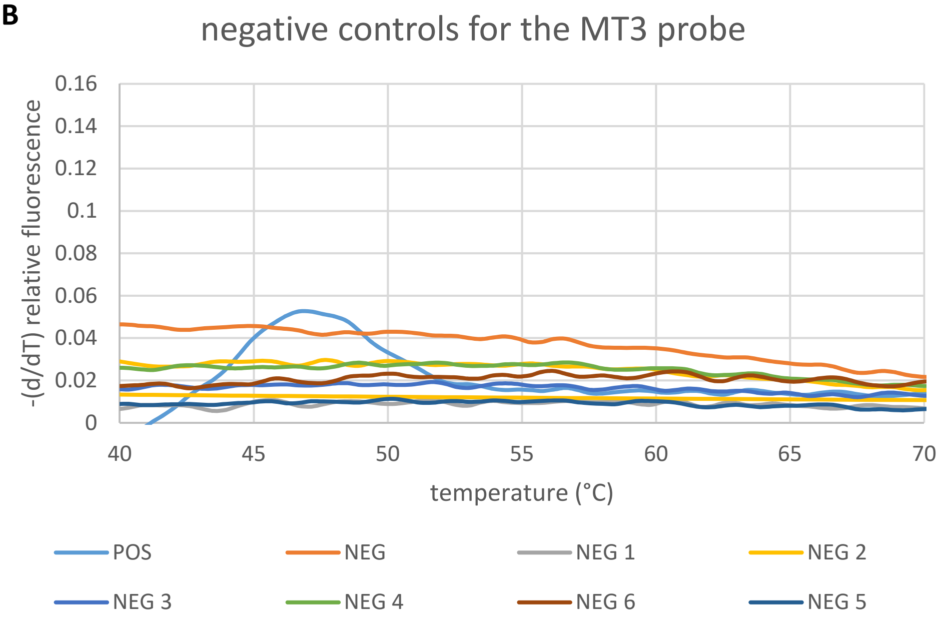

Supplement: Supplementary file 1 [file pathogens-11-00059-s001.zip › pathogens-1514952-supplementary.pdf]
